# Brugada ECG pattern: a physiopathological prospective study based on clinical, electrophysiological, angiographic, and genetic findings

**DOI:** 10.3389/fphys.2012.00474

**Published:** 2012-12-27

**Authors:** Guillaume Duthoit, Véronique Fressart, Françoise Hidden-Lucet, Françoise Simon, Darouna Kattygnarath, Philippe Charron, Caroline Himbert, Philip Aouate, Pascale Guicheney, Yves Lecarpentier, Robert Frank, Jean-Louis Hébert

**Affiliations:** ^1^Unité de Rythmologie, Institut de Cardiologie, GHU Pitié-SalpêtrièreParis, France; ^2^UF de Cardiogénétique et Myogénétique, GHU Pitié-SalpêtrièreParis, France; ^3^Faculté de Médecine Pierre et Marie Curie, Inserm-UPMC UMR S 956Paris, France; ^4^Département de Génétique, GHU Pitié-SalpêtrièreParis, France; ^5^Service de Cardiologie, Hôpital LaënnecCreil, France; ^6^Service de Physiologie Cardiorespiratoire, CHU de BicêtreLe Kremlin-Bicêtre, France

**Keywords:** Brugada syndrome, arrhythmogenic right ventricular cardiomyopathy, overlap, contrast angiography, genetic testing

## Abstract

**Introduction:** Brugada syndrome (BrS) is considered a primary electrical disease. However, morphological abnormalities have been reported and localized arrhythmogenic right ventricular (RV) dysplasia/cardiomyopathy (ARVD/C) may mimic its phenotype, raising the question of an overlap between these two conditions and making difficult the therapeutic management of patients with borderline forms. The main objective of this study was to assess prospectively the prevalence of BrS and ARVD/C on the basis of international criteria, in patients with BrS-ECG and normal echocardiography, looking for a potential overlap between the two pathologies. The secondary objectives were to describe and quantify angiographic structural alterations, hemodynamics, electrophysiology, and genetics in the setting of BrS-ECG. **Materials and Methods:** Hundred and fourteen consecutive patients matched in age underwent prospectively cardiac catheterization and quantitative biventricular contrast angiography to rule out a structural heart disease. Fifty-one patients with a BrS-ECG (BrS group, 7 F, 44 M, 43 ± 11 y) had a spontaneous or ajmaline-induced BrS coved type ECG. For angiographic comparison, 49 patients with localized ARVD/C but without ST segment elevation in the right precordial leads (14 F, 35 M, 39 ± 13 y) were also studied. They fulfilled international ESC/WHF 2000 criteria and presented angiographic localized forms, mainly confined to hypokinetic anteroapical zone (characterized by trabecular dysarray and hypertrophy), and/or diaphragmatic wall, thus resulting in RV normal volumes and preserved systolic function. These two populations were also compared with 14 control patients (7 F, 7 M, 38 ± 16 y). Among BrS group, we identified three main angiographic phenotypes: BrS group I = patients with normal RV (*n* = 15, 29%); BrS group II = patients with segmental RV wall motion abnormalities but no structural arguments for ARVD/C (*n* = 26, 51%); BrS group III = patients with localized abnormalities suggestive of focal ARVD/C (*n* = 10, 20%). **Results:** Among BrS group, 34/51 patients (67%) fulfilled BrS HRS/EHRA 2005 criteria. Nineteen (37%) were symptomatic for aborted sudden death, agonal nocturnal respiration or syncope. Ventricular stimulation was positive in 14 patients (28%). Angiography showed RV abnormalities in 36/51 patients (71%) of BrS group (BrS groups II and III). Late potentials were present in 73% (100% sensitivity and NPV for an angiographic ARVD/C, but poor specificity and PPV, both 37%). In BrS group III, 8/10 patients (16% of BrS patients) finally fulfilled international ESC/WHF 2000 ARVD/C criteria and 5/10 (10% of BrS patients) fulfilled BrS diagnostic criteria. An overlap was observed in 4 patients (8% of BrS patients) who fulfilled both ARVD/C and BrS criteria. Among the 45 genotyped patients, only one presented a *SCN5A* mutation, whereas a *TRPM4* mutation was found in another patient. Both belonged to BrS group II. *MOG1* gene analysis was negative for all patients, as were *PKP2, DSP, DSG2*, and *DSC2* analyzes performed in BrS group III. **Conclusions:** Seventy-one percent of patients with a BrS-ECG had abnormal RV wall motion and 16 had structural alterations corresponding to localized (anteroapical and/or diaphragmatic) ARVD/C. Moreover, 8% of BrS-ECG patients fulfilled both BrS and ARVD/C criteria. Our results support the hypothesis of an overlap between BrS and localized forms of ARVD/C. Conversely, genetic screening was poorly contributive for both diseases in the present series.

## Introduction

Brugada syndrome (BrS) predisposes to an increased risk of sudden cardiac death (SCD) due to ventricular fibrillation (VF) in apparently normal hearts (Brugada and Brugada, 1992). Its diagnostic criteria have been described in the second consensus conference (Wilde et al., 2002; Antzelevitch et al., 2005), where experts emphasized the need for exclusion of an organic heart disease, particularly an arrhythmogenic right ventricular (RV) dysplasia/cardiomyopathy (ARVD/C), before establishing the diagnosis of BrS. However, morphological assessment has been limited to echocardiography in most studies and has not been yet codified. Moreover, several authors showed evidence for structural, dynamic (Martini et al., 1989; Takagi et al., 2001; Papavassiliu et al., 2004), and histological (Martini et al., 1989; Corrado et al., 1996, 2001; Frustaci et al., 2005) RV abnormalities in patients with typical BrS. Brugada ECG pattern (BrS-ECG) indeed has been unmasked by ajmaline drug-challenge in 16% of 55 ARVD/C patients (Peters et al., 2004), and several authors made the hypothesis of an overlap between BrS and ARVD/C (Perez Riera et al., 2005), raising the difficult question of therapeutic management of patients with borderline forms. Two recent publications (Frustaci et al., 2005; Kim et al., 2006) showed that the “BrS” phenotype was not specific of one disease but could be the result of early, i.e., localized, organic abnormalities, underdiagnosed by echocardiography or MRI but revealed by invasive contrast angiography (microaneurysms or ARVD/C) or by endomyocardial biopsies (myocarditis, ARVD/C, cardiomyopathy).

On one hand, *SCN5A* mutation accounts for only 15–25% of patients with BrS (Ackerman et al., 2011), with an autosomal dominant inheritance (Chen et al., 1998). Other mutations have been described but appear mostly private and HRS/EHRA guidelines only recommend to restrict genetic testing to *SCN5A* in BrS (Ackerman et al., 2011). On the other hand, six genes (PKP2 = plakophillin-2, *DSP* = desmoplakin, *DSG*2 = desmoglein-2, *JUP* = junctional plakoglobin, *TGF*β 3 = Transforming Growth Factor-β3, *R*yR2 = cardiac Ryanodin Receptor-2) and 12 loci have been identified in ARVD/C so far, accounting for 30–70% of cases (Basso et al., 2006; Corrado and Thiene, 2006; Pilichou et al., 2006; Van Tintelen et al., 2006; Sen-Chowdhry et al., 2007a,b), with an autosomal dominant inheritance in half of cases (Hamid et al., 2002) and with a tropism for desmosomal proteins.

The main objective of the present study was to look for a potential overlap between BrS and ARVD/C by assessing prospectively the prevalence of these two pathologies in patients presenting in daily practice with a typical BrS-ECG and a normal echocardiography. For this purpose, we used international criteria (HRS/EHRA 2005 criteria for BrS; ESC/WHF 2000 criteria for ARVD/C) (McKenna et al., 1994; Corrado et al., 2000; Antzelevitch et al., 2005) and quantitative contrast angiography of the RV which is considered a gold standard for ARVD/C diagnosis, particularly in localized forms (Hébert et al., 2004). The secondary objectives were to describe and quantify angiographic structural alterations, hemodynamics, electrophysiology, and genetics in the setting of BrS-ECG.

## Materials and methods

### Study population

We conducted a prospective and monocentric study involving 114 patients who were matched in age and consecutively explored in our catheterization laboratory from April 2001 to July 2006 to rule out a structural heart disease. Invasive studies were performed after having obtained the written consent of each patient.

The population under study comprised two groups of patients and a control group: First, 51 patients (BrS group) were included because of a type 1 (coved-type) BrS-ECG, either spontaneously (*n* = 19) or during ajmaline test (performed in 41 patients), according to the criteria defined in the second consensus conference report (Antzelevitch et al., 2005). They had also a normal echocardiography. Second, 49 consecutive patients with localized ARVD/C and without ST segment elevation in the right precordial leads were also studied for angiographic comparison. All fulfilled ESC/WHF 2000 criteria for ARVD/C (McKenna et al., 1994; Corrado et al., 2000) and presented angiographic localized forms, mainly confined to hypokinetic anteroapical zone (characterized by trabecular dysarray and hypertrophy), and/or diaphragmatic wall, thus resulting in RV normal volumes and preserved systolic function [particularly RVEF ≥ 35% and tricuspid annulus plane systolic excursion (TAPSE) ≥14 mm]. Third, these two populations were compared with 14 control patients matched in age and consecutively recruited during the same period.

**Figure 1:**
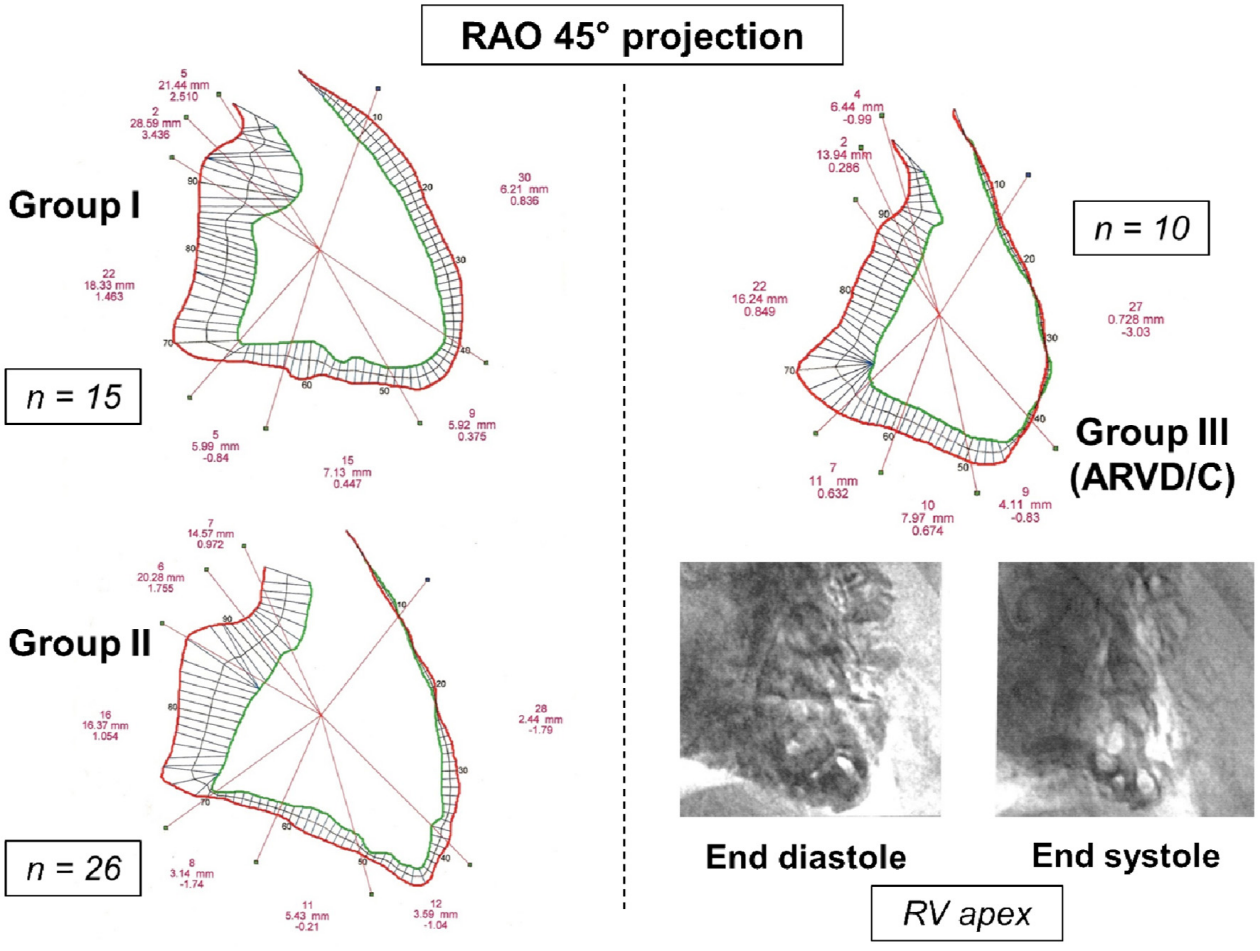
**Right ventricular (RV) angiographic contours in a 45° right anterior oblique (RAO 45°) projection and cineangiographic classification of BrS group, according to qualitative analysis of image-recordings and to quantitative analysis of right ventricular segmental excursion analysis**. BrS group I: normal RV (patient 32); BrS group II: patients with BrS-ECG and segmental RV wall motion abnormalities but without arguments for ARVD/C (patient 40: anterior RV akinesia, apical, inferior and subtricuspid hypokinesia, normal TAPSE, and crista supraventricularis shortening); and BrS group III: patients with BrS-ECG and angiographic abnormalities compatible with ARVD/C (patient 42: anteroapical akinesia, trabecular disarray and hypertrophy, end systolic polycyclic images). For each contour, the segmental excursions between diastole and systole are expressed in purple characters (from top to bottom: number of analyzed chords for each segment; mean segmental excursion in mm; standard deviation from normal).

Among BrS group, we identified three main phenotypes (Figure 1): (1) BrS group I (or “pure” BrS) corresponding to patients with BrS-ECG with strictly normal RV; (2) BrS group II corresponding to patients with BrS-ECG and segmental RV wall motion abnormalities but without arguments for ARVD/C; and (3) BrS group III corresponding to patients with BrS-ECG and angiographic structural abnormalities compatible with ARVD/C. For all of them, underlying structural cardiac abnormalities had been excluded previously by physical examination, chest X-ray, 2-dimensional echocardiography, and by stress-ECG when appropriate. All patients had normal serum [K^+^] and [Ca^2+^], troponin I_c_, C-Reactive Protein, and TSH_us_ at the time of diagnosis.

Control patients (7 females, 7 males) had been explored consecutively during the same period without medication in our unit for atypical chest pain associated with a modified stress-ECG (10 patients) or persistant suspicion of pulmonary embolism despite normal scintigraphy, finally excluded by pulmonary contrast angiography (4 patients). All had normal pressures, RV and LV volumes and coronary angiograms. Quantitative data concerning their hemodynamics and segmental excursion were used to define normal values and reference nomograms for segmental excursion [in both 45° right anterior oblique (RAO) and left anterior oblique (LAO) projections for each ventricle].

Ajmaline was administered under careful monitoring (1 mg/kg body weight, at a rate of 1 mg/s with a maximal dose of 100 mg). Patients were excluded if a QRS widening ≥30% occurred even associated with a coved-type ECG, unless the type 1 pattern remained present in two right precordial leads (V_1_, V_2_, or V_3_) after QRS width normalization. All patients actually received a dose of 1 mg/kg of ajmaline. At the rate of 1 mg/s, conduction slowing and/or ventricular proarrhythmic effect appear in our experience between 2 and 4 min after starting the infusion and never before 90 s. This means that the full dose of ajmaline (max 100 mg) is actually given when its effects begin on ECG.

A proband was defined as the first case of Brugada ECG diagnosed within the family. Familial history of SCD, BrS, or ARVD/C was defined accordingly to international criteria (McKenna et al., 1994; Antzelevitch et al., 2005). Family history of BrS was defined by the presence of one of the following findings documented at the time of inclusion: sudden cardiac death of a first degree relative under the age of 45 years; documented coved-type ECG in family members (spontaneous or induced).

### Electrophysiology (BrS group)

Thirty-seven patients underwent signal-averaged ECG (SA-ECG, high-pass filter of 25 Hz and/or 40 Hz, low-pass filter of 250 Hz). Ventricular late potentials were defined by the presence of ≥2 of the 3 ESC/AHA/ACC criteria (filtered QRS > 114 ms at 40 and 25 Hz; LAS > 38 ms at 40 Hz, LAS ≥ 32 ms at 25 Hz; RMS40 < 20 μV at 40 Hz, RMS40 ≤ 25 μV at 25 Hz) (Gomes et al., 1987; Breithardt et al., 1991).

Electrophysiological study, performed in all but one patient (*n* = 50), included basal measurement of HV interval (*n* = 36) and programmed ventricular stimulation (PVS, *n* = 50) without antiarrhythmic drugs. PVS had to be performed after angiography during the same hospitalization, or at least one month before angiography to avoid functional wall motion abnormalities due to fast pacing or recent electrical cardioversion. PVS was applied at two sites [apex and RV outflow tract (RVOT)], at two drive cycle lengths (600 and 400 ms), using a maximum of three extrastimuli with a minimum coupling interval of 200 ms. Patients were inducible if a sustained (>30s) or syncopal ventricular arrhythmia occurred [VF, polymorphic ventricular tachycardia (VT), or monomorphic VT (Priori et al., 2002; Antzelevitch et al., 2005)].

### Non-invasive imaging (BrS group)

Among BrS group, all included patients had normal 2-dimensional echocardiography. Mean LVEF was 64 ± 5% in BrS group. Moreover, 32 patients underwent biventricular phase angioscintigraphy and 29 patients underwent cardiac magnetic resonance imaging (MRI).

### Cardiac catheterization

All patients underwent right and left heart catheterization, and coronary angiography performed by the same operator. Fluid-filled pressures and pulmonary artery thermodilution cardiac output were assessed (Swan-Ganz catheter, Abbott, Chicago, Illinois). RV angiography was performed through a 145° 6F angled pigtail catheter positioned at the RV apex, beyond the moderator band. Left heart catheterization was performed through a straight 6F pigtail catheter (Cordis, Roden, The Netherlands). All patients had normal coronary angiograms.

RV and LV cineangiograms were recorded using two successive orthogonal views (RAO45° and LAO45° without angulation). We chose these specific projections for better measurement of TAPSE and crista supraventricularis shortening in the RAO projection and septal morphology in LAO projection, whatever RV dilation (Hébert et al., 2004). Image calibration used a 40 mm metal ball. Images were analyzed by two independent trained cardiologists (JLH, GD) using dedicated software (Philips Research Laboratory, Suresnes, France).

Based on ESC/WHF 2000 criteria (Marcus et al., 1982; McKenna et al., 1994; Corrado et al., 2000) and Hébert et al. work (Hébert et al., 2004), RV structural abnormalities were defined by hypertrophic trabeculae ≥4 mm (“deep fissuring” of the anterior wall), areas of negative contrast in the trabecular zone and/or the moderator band (polycyclic images), apical aneurysm often associated with Y-shaped negative contrast image, large bulging or multiple outpouchings of the diaphragmatic wall, diastolic bulging of the subtricuspid area, RVOT aneurysm as well as RV free wall bulging in LAO projection.

**Figure 2:**
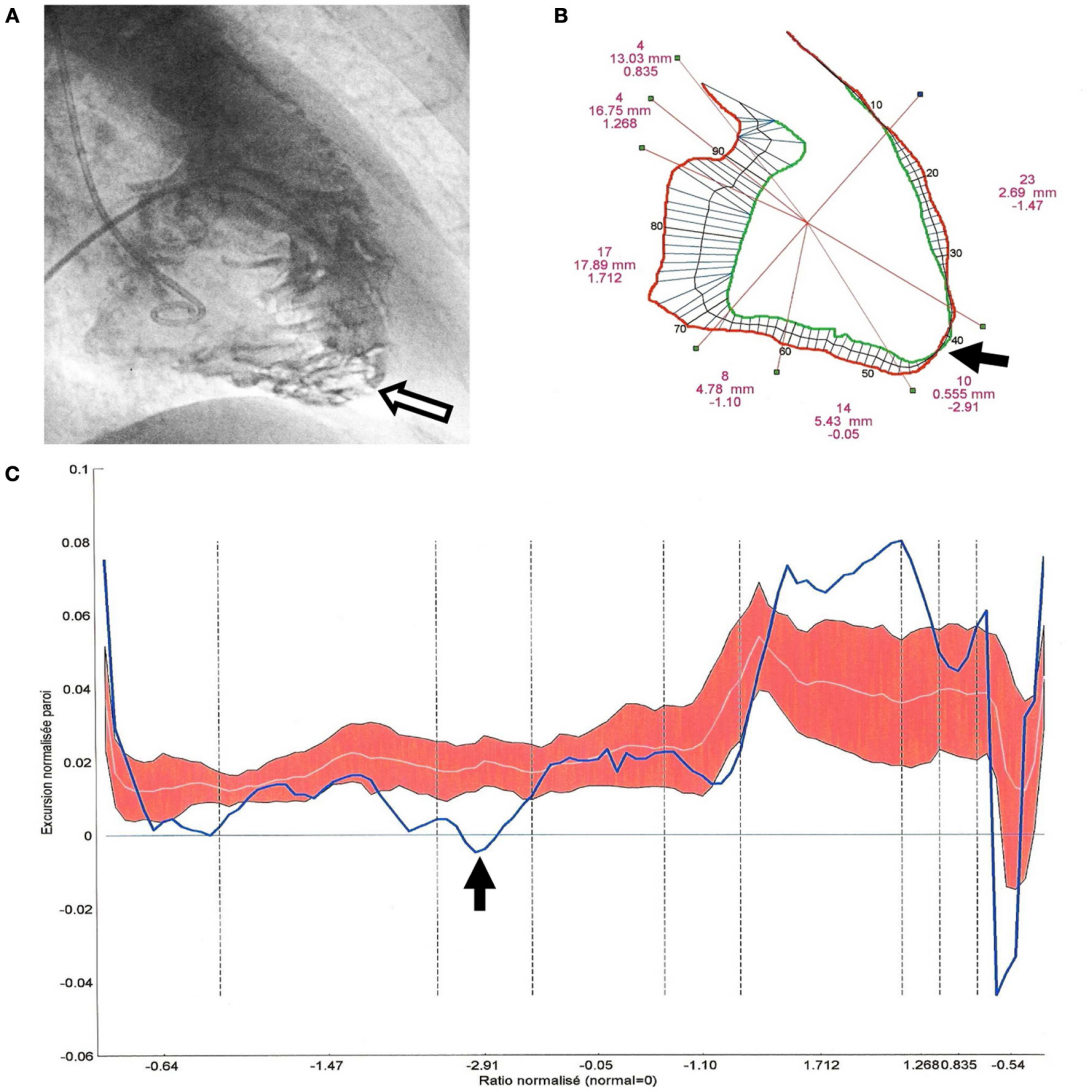
**Quantitative angiographic analysis of segmental right ventricular (RV) excursions in a 45° right anterior oblique projection (patient 43, BrS group III, ARVD/C with BrS-ECG). (A)** RV cineangiogram at end diastole: trabecular hypertrophy (>4 mm) and disarray which is predominant at the apical part of the RV (white arrow). **(B)** RV end diastolic (red) and end systolic (green) contours showing an akinetic infundibulotrabecular junction (i.e., high RVOT), a hypokinetic anterior wall, a dyskinetic apex (black arrow), a normal inferior contraction and a hypokinetic sub-tricuspid segment. TAPSE, RV volumes, and RVEF were normal. **(C)** Segmental excursions are plotted (blue line, bold) on a nomogram in a clockwise direction from the high RVOT to the TAPSE and the crista supraventricularis. Normal excursion values are represented by the red zone (the white line represents the mean values of control patients and the red zone corresponds to +1 SD or −1 SD).

Biventricular segmental motion was assessed by a modified quantitative Sheehan centerline method (Hébert et al., 2004) (100 clockwise chords, segmental shortening expressed in mm in both projections). For each projection, segmentation of RV and LV was manually performed and corresponded to a well-defined anatomical zone (Figures 1, 2, 4). The mean excursion was then automatically averaged for each segment and expressed in millimeters ± one standard deviation (SD), and transposed on a comparative nomogram for each projection, showing individual chord shortening compared to mean values (mean ± 1 SD) obtained in control patients (Figure 2). Biplane volume calculations (mL) were performed according to Boak's hemielliptical model for the RV, and to Dodge's ellipsoidal model for the LV (Hébert et al., 2004).

### Genetic analysis

DNA was extracted from peripheral blood lymphocytes according to standard procedures, after having obtained the written consent of the patients. *SCN5A* gene analysis was performed in 35 patients from BrS groups I and II (without ARVD/C). All BrS group III patients underwent a more extensive genetic study, which comprised analysis of *SCN5A* but also of genes coding for desmosomal proteins, including *PKP2*, *DSP*, *DSG2*, and *DSC2*. We did not analyze *RyR2*, *JUP*, and *TGF*β*3* with respect to the clinical presentation of the patients (absence of catecholaminergic VT or palmo-plantar hyperkeratosis), and because of the controversial role of *RyR2* in the pathogenesis of ARVD/C.

As *MOG1* (Multi-copy suppressor Of GSP1 mutants) was recently described as a partner of Na(v)1.5, playing a potential role in the regulation of its expression and trafficking (Kattygnarath et al., 2011), we decided to study the prevalence of *MOG1* mutation in our population. Mutations in the *TRPM4* gene (Transient Receptor Potential channel (type 4) belonging to the Melastatin subfamily). *TRP* channels have been identified as a cause of an autosomal dominant form of cardiac conduction disease. As BrS patients or relatives often present with conduction disturbances (atrio-ventricular block, sick sinus syndrome, fascicular block), we decided at the time of the study to consider also *TRPM4* as a candidate gene for BrS (Stallmeyer et al., 2012).

All coding exons of the genes *SCN5A*, *MOG1*, *TRPM4* and *PKP2, DSP, DSG2, DSC2* were amplified by polymerase chain reaction (PCR) with specific primers and directly sequenced in both strands. Direct sequencing was performed with the use of the Big Dye dideoxy-terminator chemistry (Perkin Elmer) on ABI 3830 DNA sequencer (PE Applied Biosystems, Foster, CA). A mutation was defined by the presence of an abnormal variant which has not been described in previously published series, which was absent in a panel of 300 controls (600 alleles) and which was located in a highly conserved region through animal species. When available, cosegregation within the family was considered as the last clue to confirm a mutation.

### Statistical analysis

Quantitative data are expressed as mean ± SD and percentages. We used a Student's unpaired *t*-test for comparison of quantitative data and an exact Fischer's test for percentages. For multiple comparisons we performed a variance analysis (ANOVA), followed by a Student's unpaired *t*-test, and balanced by the Bonferroni correction. Qualitative data were compared using χ^2^ test. A *p*-value <0.05 was considered significant.

## Results

### Population

We included 51 patients in the BrS group (7 females, 44 males, mean age 43 ± 11 years), 49 patients with localized ARVD/C and no ST-segment elevation in the right precordial leads (14 females, 35 males, mean age 39 ± 13 years), and 14 control patients (7 females, 7 males, mean age 38 ± 16 years). The main results regarding the BrS group population are summarized in Tables 1, 2. Sex ratio M/F was significantly higher in the BrS group compared to patients with localized ARVD/C without ST abnormalities and to control patients (respectively 6.3 vs. 2.5 and 1, *p* = 0.0001, Table 3). This difference remained significant when comparing localized ARVD/C without ST abnormalities, either with a BS population pooling together BrS group I and BrS group II (i.e., BrS-ECG and no ARVD/C) or with the BrS group III (sex ratios M/F respectively of 2.5 vs. 5.8 and 9, *p* = 0.0001).

**Table 1:** **Results of investigations carried out in 41 patients with a BrS-ECG without angiographic arguments for ARVD/C (BrS groups I and II)**.

**Patient**	**Sex**	**Age**	**ECG**			**Familial history of BrS/SCD under 45 years**	**Symptoms**	**Baseline PVS**	**BrS diagnostic criteria fulfilled (HRS/EHRA 2005)**	**Angiography**	**Genetics**			
			**Baseline Brugada type**	**Coved type with ajmaline**	**Late potentials**		Sync/nocturnal agonal respiration/SCD^*^	**Induced arrhythmia**		**Segmental RV wall motion abnormalities**	**SCN5A mutation**	**Desmosomal genes**	**MOG1 mutation or TRPM4 mutation**	
1	M	27	1	+	+	−	+	−	+	−	−	na	−	***BrS group I***
2	M	40	2	+	−	−	−	SVF	+	−	−	na	−	
3	M	49	3	+	−	−	+	−	+	−	−	na	−	
4	M	32	2	+	na	−	+	NS PVT	+	−	−	na	−	
5	M	33	2	+	−	+	−	−	+	−	−	na	−	
6	M	48	3	+	+	−	+	NS PVT	+	−	na	na	na	
7	F	46	2	+	na	+	−	−	+	−	−	−	−	
8	M	51	2	+	+	+	+^*^	SVF	+	−	na	na	na	
9	M	59	1	+	+	+	−	NS PVT	+	−	−	na	−	
10	M	39	2	+	−	−	+	−	+	+	−	na	na	***BrS group II***
11	M	45	1	na	na	−	−	SVF	+	+	−	na	−	
12	M	28	3	+	na	+	−	sync NS VF	+	+	−	na	−	
13	M	35	3	+	−	−	+	−	+	+	−	na	−	
14	F	76	3	+	+	−	+	−	+	+	na	na	na	
15	M	58	1	na	na	−	+	SVF	+	+	−	na	−	
16	M	60	1	+	−	−	+	NS PVT	+	+	na	na	na	
17	M	41	2	+	+	−	+	sync NS VF	+	+	na	na	na	
18	F	56	2	+	+	−	+	−	+	+	−	na	−	
19	M	37	1	+	+	−	−	SVF	+	+	−	na	−	
20	M	21	1	+	+	+	−	SVF	+	+	−	na	−	
21	M	48	1	+	−	+	+	SVF	+	+	−	na	−	
22	F	24	3	+	−	−	−	SVF	+	+	−	na	−	
23	M	42	2	+	na	−	+	−	+	+	−	na	−	
24	M	28	2	+	na	+	+	−	+	+	-	*polym PKP2*	-	
25	M	52	1	na	+	−	+^*^	−	+	+	−	na	−	
26	F	57	2	+	na	+	+	−	+	+	−	na	−	
27	M	37	1	na	+	−	−	SVF	+	+	−	na	−	
28	F	46	1	+	na	+	−	−	+	+	−	na	−	
29	M	34	2	+	+	−	+	−	+	+	−	−	−	
30	M	45	2	+	na	−	−	−	−	−	−	na	−	***BrS group I***
31	M	34	2	+	+	−	−	−	−	−	−	na	−	
32	M	50	2	+	−	−	−	−	−	−	−	na	−	
33	M	54	1	na	na	−	−	−	−	−	***polym R481W***	*polym DSC2/polym DSG2*	-	
34	M	38	2	+	na	−	−	−	−	−	−	na	−	
35	M	49	1	na	+	−	−	−	−	−	−	na	−	
36	M	32	2	+	−	−	−	−	−	+	na	na	na	***BrS group II***
37	M	49	1	+	na	−	−	−	−	+	−	na	**TRPM4 (Q131H)**	
38	M	33	1	na	+	−	−	−	−	+	−	na	na	
39	M	40	2	+	na	−	−	−	−	+	−	na	−	
40	M	47	2	+	+	−	−	NS PVT	−	+	−	na	−	
41	M	45	1	+	+	−	−	−	−	+	**R965C/ex 17.1**	−	−	

*On white background = patients with Brugada syndrome according to HRS/EHRA guidelines. On a gray background = patients with a BrS-ECG but with no sufficient data to validate HRS/EHRA criteria for Brugada syndrome*.

*Bold entries for results of genetic screening correspond to the presence of a confirmed mutation OR to the presence of a special polymorphism which might not be benign*.

*Bold italic entries for “BrS group I” delineates patients 1–9 and 30–35. “BrS group II” delineates patients 10–29 and 36–41*.

Abbreviations + *present* − *absent* *na* *not available* *BrS* *Brugada syndrome* SCD or^*^ *Sudden cardiac death* *SVF* *sustained ventricular fibrillation* *NS PVT* *non-sustained polymorphic ventricular tachycardia* *sync* *syncope or syncopal* *RV* *right ventricular* *polym* *polymorphism*.

**Table 2:** **Results of investigations achieved in 10 patients belonging to BrS group III (BrS-ECG with angiographic ARVD/C)**.

**Patient**	**Sex**	**Age**	**Familial history of BrS/SCD under 45 years**	**Familial history of ARVD/C**		**Symptoms**	**ECG**			**Arrhythmias**		**Baseline PVS**	**Angiography**	**ARVD/C diagnostic criteria fulfilled (ESC/WHF 2000)**	**BrS diagnostic criteria fulfilled (HRS/EHRA 2005)**	**Genetics**		
				**Minor**	**Major**		**Baseline Brugada type**	**Type 1 with ajmaline**	**Late potentials**	**VT with LBBB morphology**	**PVC >1000/24h**	**Induced arrhythmia**	**RV wall motion abnormalities**	**Positive diagnosis if: ≥2M or 1M/2m or 4m**		**SCN5A mutation**	**Desmosomal genes**	**MOG1 or TRPM4 mutation**
42	F	48	−	−	−	Lipothymia	3	+	+	NS MVT	+	NS PVT	anteroapical akinesia	YES, 1M/4m	+	−	−	−
43	M	51	−	−	−	−	1	+	+	−	−	SVF	anteroapical hypokinesia	YES, 1M/2m	+	−	rare *polym PKP2* (A264V)	−
44	M	36	−	−	−	Chest pain	1	na	+	−	−	−	anterior akinesia, apical hypokinesia	NO, 1M/1m	−	−	*polym DSG2* (S882P)	−
45	M	50	SCD	+	−	Lipothymia	1	na	+	−	−	SVF	anteroapical hypokinesia, apical amputation	YES, 1M/2m	+	−	−	−
46	M	58	−	−	−	Syncope	2	+	+	−	−	SVF	anteroapical akinesia	NO, 1M/1m	+	−	−	na
47	M	41	−	−	−	Chest pain	2	+	+	−	−	−	anteroapical & inferior hypokinesia, subtricuspid akinesia	YES, 2M/2m	−	−	−	−
48	M	54	−	−	−	−	2	+	+	−	+	na	anteroapical dyskinesia, inferior hypokinesia	YES, 1M/3m	−	−	−	na
49	M	37	−	suspect	−	−	2	+	+	−	+	−	anteroapical hypokinesia	YES, 1M/3m	−	−	−	−
50	M	36	SCD	+	+	−	3	+	+	−	−	−	anteroapical hypokinesia	YES, 2M/3m	+	−	−	−
51	M	25	−	−	−	Lipothymia	2	+	+	−	−	−	anteroapical akinesia	YES, 2M/2m	−	−	−	−

*Clinical presentation, ECG, electrophysiological data; qualitative data from contrast angiography; International criteria for ARVD/C or Brugada syndrome; results of genetic screening*.

Abbreviations + *present* − *absent* *BrS* *Brugada syndrome* *SCD* *sudden cardiac death* *na* *not available* *VT* *ventricular tachycardia* *LBBB* *left bundle branch block* *NS MVT/PVT* *non-sustained monomorphic/polymorphic VT* *SVF* *sustained ventricular fibrillation* *RV* *right ventricular* *Criteria M/m* *Major/minor* *polym* *polymorphism*.

**Table 3:** **Data from hemodynamic and angiographic quantitative study: patients with a BrS-ECG vs. localized ARVD/C without ST syndrome and vs. control patients**.

	**BrS Group I (*n* = 15)**	**BrS Group II (*n* = 26)**	**BrS Group III (*n* = 10)**	***P*-value**	**Whole BrS population (*n* = 51)**	**Localized ARVD/C without ST syndrome (*n* = 49)**	**Control patients (*n* = 14)**	***P*-value**
Age (years)	44 ± 9	43 ± 13	44 ± 10	NS	43 ± 11	39 ± 13	38 ± 16	NS
Sex ratio H/F	14 (14/1)	4.2 (21/5)	9 (9/1)	NS	6.3 (44/7)	2.5 (35/14)	1 (7/7)	0.0001^**^^◊^
Body Surface Area (m^2^)	1.8 ± 0.1	1.9 ± 0.2	1.9 ± 0.2	NS	1.9 ± 0.2	1.8 ± 0.2	1.8 ± 0.2	NS
Heart Rate (bpm)	75 ± 12	78 ± 15	75 ± 13	NS	76 ± 14	69 ± 14	77 ± 13	0.009^**^
Cardiac Index (L/min/m^2^)	3.8 ± 0.6	3.9 ± 0.9	3.8 ± 0.7	NS	3.9 ± 0.8	3.3 ± 0.8	3.4 ± 0.7	0.003^**^
PVR (dynes.s.cm^-5^)	76 ± 28	70 ± 29	74 ± 41	NS	72 ± 31	81 ± 43	92 ± 48	NS
SVR (dynes.s.cm^-5^)	1175 ± 254	1104 ± 359	1044 ± 193	NS	1113 ± 302	1239 ± 368	1285 ± 436	NS
AVO_2_CD (vol%)	4.1 ± 0.6	3.8 ± 0.6	4 ± 0.6	NS	4.0 ± 0.6	4.0 ± 0.9	3.9 ± 1.4	NS
RVEDV_i_ (mL/m^2^)	86 ± 19	81 ± 17	86 ± 14	NS	83 ± 17	82 ± 20	85 ± 23	NS
RVESV_i_ (mL/m^2^)	36 ± 11	35 ± 10	38 ± 10	NS	36 ± 10	38 ± 11	37 ± 11	NS
RV Stroke index (mL/m^2^)	50 ± 10	45 ± 8	48 ± 6	NS	47 ± 9	44 ± 11	48 ± 14	NS
RVEF (%)	59 ± 5	57 ± 6	56 ± 5	NS	57 ± 5	54 ± 5	58 ± 5	0.006^**^
**SEGMENTAL EXCURSIONS**								
Anterior RV (mm)	6 ± 1	2 ± 2	2 ± 2	<0.001^*^^,^^†^	3 ± 2	2 ± 2	5 ± 1	<0.0001^**^^◊^^♦^
RV Apex (mm)	6 ± 3	2 ± 2	2 ± 2	<0.0001^*^, 0.006^†^	3 ± 3	2 ± 2	5 ± 2	0.0007^**^^◊^^♦^
Inferior RV (mm)	7 ± 3	5 ± 1	6 ± 2	0.01^*^	6 ± 2	6 ± 2	7 ± 2	NS
Sub-tricuspid RV (mm)	7 ± 3	6 ± 2	6 ± 3	NS	6 ± 2	7 ± 3	8 ± 3	0.04^◊^
TAPSE (mm)	15 ± 4	17 ± 4	17 ± 2	NS	16 ± 3	15 ± 3	16 ± 2	0.03^**^
Crista supraventricularis (mm)	14 ± 3	15 ± 3	15 ± 3	NS	15 ± 3	13 ± 3	14 ± 4	0.04^**^
High RVOT (mm)	9 ± 3	9 ± 4	9 ± 4	NS	9 ± 4	6 ± 3	7 ± 2	0.001^**^
Mid-lateral RV (mm)	13 ± 3	13 ± 4	13 ± 4	NS	13 ± 3	11 ± 4	10 ± 3	0.0007^**^
LVEDV_i_ (mL/m^2^)	77 ± 13	73 ± 15	77 ± 19	NS	75 ± 15	73 ± 19	72 ± 20	NS
LVESV_i_ (mL/m^2^)	23 ± 6	25 ± 8	24 ± 11	NS	25 ± 8	27 ± 10	25 ± 8	NS
LV Stroke index (mL/m^2^)	53 ± 9	47 ± 9	53 ± 10	NS	50 ± 10	46 ± 13	47 ± 14	NS
LVEF (%)_	70 ± 5	65 ± 6	69 ± 7	0.03^*^ (trend)	67 ± 6	63 ± 8	64 ± 6	0.01^**^
**SEGMENTAL EXCURSIONS**								
Anterior LV (mm)	11 ± 2	10 ± 2	10 ± 3	NS	10 ± 2	10 ± 3	10 ± 2	NS
Inferior LV (mm)	11 ± 2	10 ± 2	10 ± 4	NS	10 ± 2	9 ± 3	9 ± 3	NS
Lateral LV (mm)	10 ± 2	8 ± 2	10 ± 3	0.02^*^^‡^ (trend)	9 ± 2	9 ± 3	9 ± 3	NS

*BrS Group I: BrS-ECG and normal RV; BrS Group II: Brugada ECG and segmental RV wall motion abnormalities with no arguments for ARVD/C; BrS Group III: Brugada ECG and angiographic abnormalities compatible with ARVD/C (trabecular disarray)*.

Left: Unpaired t-test, p significant after Bonferroni correction if ≤0.016:

* BrS Group I vs. BrS Group II;

† BrS Group I vs. BrS Group III;

‡ *BrS Group II vs. BrS Group III*.

Right: Factorial ANOVA, p significant if < 0.05:

** group with BrS-ECG vs. group with localized ARVD/C;

◊ group with BrS-ECG vs. control group;

♦ *group with localized ARVD/C vs. control group*.

Abbreviations *PVR/SVR* *pulmonary/systemic vascular resistances* *RV/LV* *right/left ventricle* *AVO_2_CD* *arteriovenous oxygen content difference* *EDVi/ESVi* *end-diastolic/end-systolic volume index* *EF* *ejection fraction* *TAPSE* *tricuspid annular plane systolic excursion* *RVOT* *right ventricular outflow tract*.

Among BrS group, 19/51 patients (37%) presented with a spontaneous BrS type 1 ECG. The remaining 32 had a baseline BrS type 2 or 3 ECG, converted to type 1 under ajmaline infusion. Familial screening of BrS concerned only 5 patients of our series, whereas the remaining 46 were probands. Nineteen patients (37%) were symptomatic for aborted sudden death, agonal nocturnal respiration or syncope. Two patients presented with aborted SCD (documented nocturnal VF), 2 others with nocturnal seizures and syncope history, and 15 with at least one syncopal episode. Among patients without aborted sudden death, agonal nocturnal respiration or syncope, 2 patients presented palpitations and dizziness, 6 complained of lipothymia, and 5 others of palpitations. Remarkably, 12/34 patients (24%) had atypical chest pain, which was the cause of achieving an ECG in 10 patients. For the remaining 9 asymptomatic patients (18%), BrS-ECG diagnosis was fortuitous (systematic ECG). At the time of inclusion, 3 patients had an ICD because of type 1 ECG associated with history of aborted SCD, nocturnal agonal respiration or syncope. There was no statistical difference in terms of symptoms (syncope, SCD, seizures) between patients regarding the BrS-ECG type (1, 2, or 3) (*p* = 0.5). Moreover, no statistical significant difference was observed between BrS groups I, II, and III, in terms of symptoms (atypical chest pain, syncope or pre-syncope, seizures, SCD, *p* = 0.08).

### Primary outcome (tables 1, 2)

Among BrS group, 34/51 patients (67%) fulfilled BrS HRS/EHRA 2005 criteria (Antzelevitch et al., 2005), mainly because 19/51 (37%) were symptomatic with aborted sudden death, agonal nocturnal respiration or syncope, because of a positive ventricular stimulation in 14 patients (28%) or because of a familial history of SCD before the age of 45 years in 10 patients (20%). Among BrS group III, 8/10 patients (16% of all the BrS-ECGs) fulfilled international ESC/WHF 2000 ARVD/C criteria (McKenna et al., 1994; Corrado et al., 2000) and 5/10 (10% of all the BrS-ECGs) fulfilled BrS HRS/EHRA 2005 diagnostic criteria. An overlap was observed in 4 patients (8% of all the BrS-ECGs) who fulfilled both ARVD/C and BrS international criteria.

### Non-invasive electrophysiology (BrS group)

**Figure 3:**
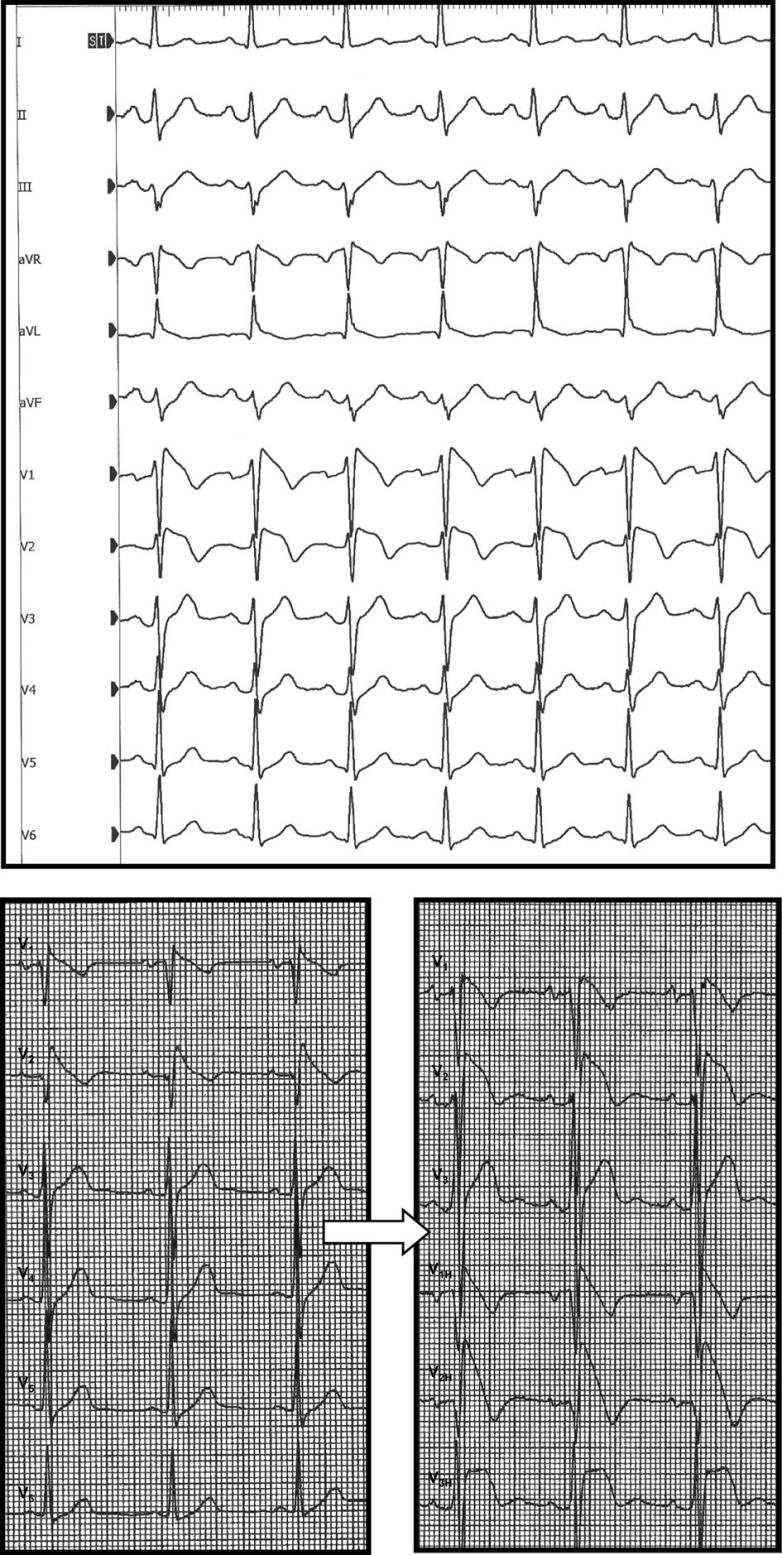
**Two typical BrS-ECGs (speed 25 mm/s; gain 1 mm/mV)**. **Top:** patient 33 with a baseline type 1 Brugada ECG (BrS group I). **Bottom:** patient 43 with a spontaneous type 1 Brugada ECG (left panel), becoming caricatural after ajmaline infusion (right panel, BrS group III, ARVD/C). V_1H_, V_2H_, V_3H_ mean upper space above the right precordial leads V_1_, V_2_, and V_3_, respectively.

Systematic ECG recording in one upper space above the usual right precordial leads (3rd intercostal space for V_1H_ (V_1_ “high”) and V_2H_, 4th intercostal space for V_3H_) showed a BrS type 1 ECG in 63% of the cases (*n* = 26, Figure 3) and allowed the diagnosis of BrS-ECG in 34% (*n* = 14, where a coved-type pattern was only visible in one of the classical leads V_1_, V_2_, V_3_, but also in one or more of the leads V_1H_, V_2H_, V_3H_). Reversible conduction slowing was observed in 24% of the cases (*n* = 10, mainly PR interval and QRS duration (>30%) prolongation). No further complication occurred.

Baseline ECG showed a prolonged PR interval (≥200 ms) in 7 patients (14%), a left anterior fascicular block (QRS axis beneath −30°) in 7 patients (14%) and a left posterior fascicular block in one patient (patient 28, BrS group II, with a PR interval of 200 ms). Remarkably, a left anterior fascicular block was present in 2 patients with ARVC/D (BrS group III) as compared to 5 patients (*p* = NS) without ARVD/C [BrS (I+II) groups]. A prolonged PR interval alone was present in 2 patients with ARVD/C (BrS group III) and in 4 patients without ARVD/C [BrS group (I+II), *p* = NS].

Holter-ECG or continuous monitoring showed premature ventricular contractions in 12/51 patients (24%), with a left bundle branch block pattern in 9, and associated with non-sustained RVOT tachycardia in one patient from BrS group III (*n* = 42).

SA-ECG was performed in 37/51 patients from BrS group (73%) at 40 and/or 25 Hz. When combining the results of SA-ECG at 40 and/or 25 Hz, late potentials were present in 27 patients out of 37 (73%). The presence of late potentials (at 40 and/or 25 Hz) was associated with a sensitivity of 100%, a specificity of 37%, a positive predictive value (PPV) of 37% and a negative predictive value (NPV) of 100% for angiographic ARVD/C diagnosis (BrS group III). Late potentials were significantly more frequent at 40 Hz and at 40 and/or 25 Hz in BrS group III compared to patients with a BrS-ECG and no ARVD/C [BrS groups (I+II), χ^2^ test, *p* = 0.04 and *p* = 0.02 respectively, *p* = 0.06 for 25 Hz alone].

### Invasive electrophysiological study (BrS group)

Mean HV interval was 48 ± 7 ms (range 38–65 ms). Mean RV effective refractory period was 226 ± 20 ms. A sustained/syncopal VF or polymorphic VT was induced in 28% of the cases (*n* = 14), while non-sustained polymorphic VT was induced in 7 additional patients. We found no difference concerning inducibility between BrS groups I, II, and III, neither between asymptomatic and symptomatic patients, nor between patients with or without spontaneous type 1 BrS-ECG. Finally, one of the 2 patients who experienced previous SCD was not inducible (*n* = 25).

### Non-invasive imaging (BrS group)

Cardiac MRI was performed in 29 BrS patients. Overall, MRI abnormalities (mainly localized RV wall motion abnormalities) were seen in 45% (13/29) of BrS patients, with fatty infiltration in 17% (5/29), which was often considered as non-specific by radiologists.

Radionuclide angiography was performed in 32 BrS patients. Mean RVEF was 52 ± 7% and mean LVEF was 64 ± 5% (versus contrast angiography, *p = NS and p* = 0.03, respectively). We noticed in 25% of the 32 explored cases (*n* = 8) a RVOT phase delay and a localized RV hypokinesia in 13% (*n* = 4). Seven patients from BrS group III underwent angioscintigraphy, which confirmed in two cases the diagnosis of ARVD/C made with contrast angiography (marked localized hypokinesia associated with a RVOT phase delay). Despite a poor sensitivity for ARVD/C diagnosis, angioscintigraphy had an excellent PPV (100%) and a good NPV (86%) as compared to contrast angiography (Table 3, bottom).

### Cardiac catheterization

#### Hemodynamical study (table 3)

All groups under study were comparable in terms of right or left heart pressures, pulmonary or systemic vascular resistances (PVR, SVR), arteriovenous oxygen content difference, or hemoglobin content. The significant difference in heart rate observed between BrS group and patients with localized ARVD/C without ST segment elevation was attributed to the frequent prescription of β-blockers in the last group (76 ± 14 vs. 69 ± 14 bpm, *p* = 0.009). Cardiac index was significantly higher in patients from the BrS group compared to patients with localized ARVD/C without ST abnormalities (3.9 ± 0.8 vs. 3.3 ± 0.8 L/min/m^2^, *p* = 0.003). This was also the case when comparing BrS group III (ST-syndrome and ARVD/C) with localized ARVD/C without ST abnormalities (3.9 ± 0.7 vs. 3.3 ± 0.8 L/min/m^2^, *p* = 0.01). This finding could be also attributed to the lower heart-rate of this group. We observed no significant difference in terms of cardiac index between BrS groups I, II, III, and control patients.

#### Digitized quantitative contrast angiography

**Figure 4:**
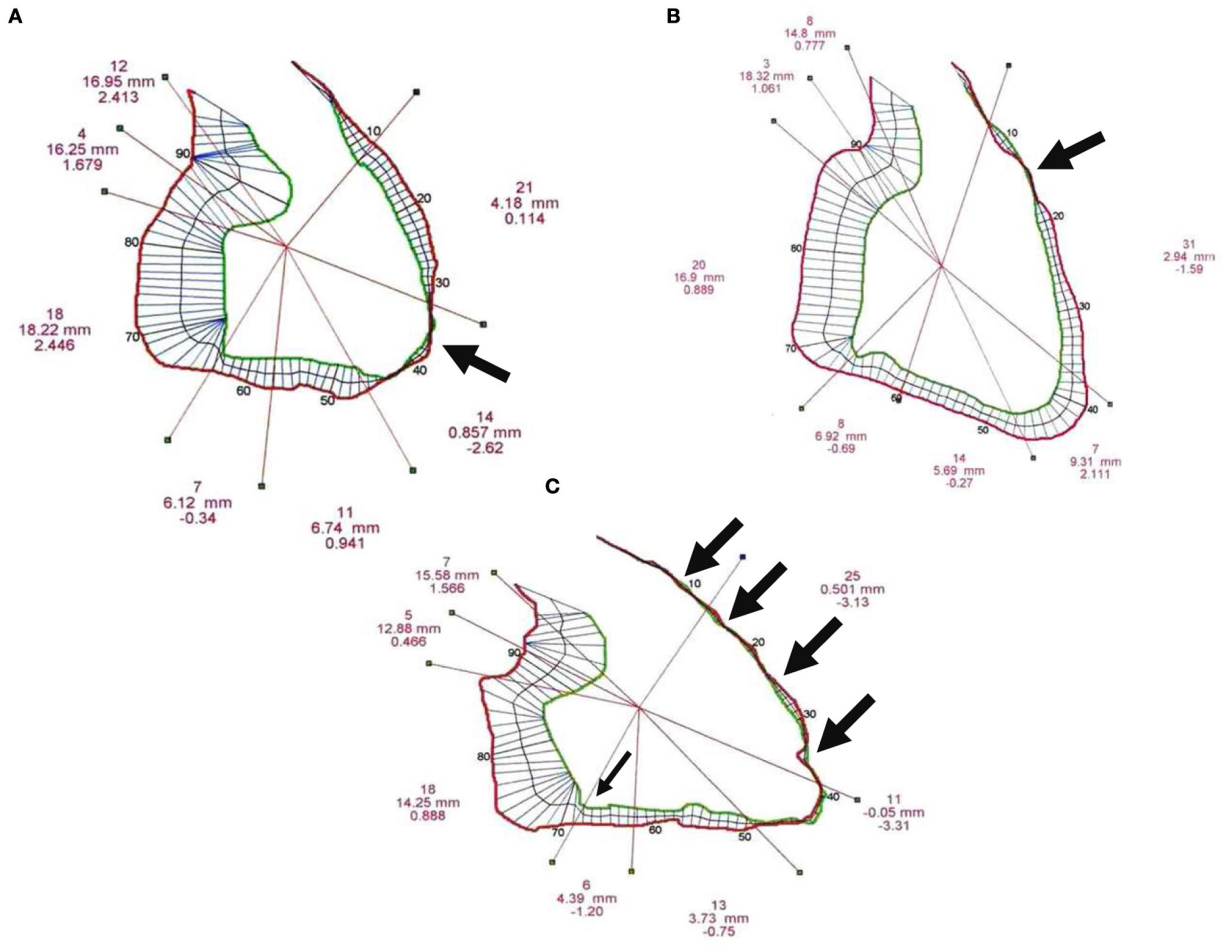
**Three typical examples of right ventricular (RV) segmental wall motion abnormalities observed in BrS group II**. **(A)** Akinesia restricted to the RV apex (bold black arrow). **(B)** Hypokinesia or outpouching without dyskinesia of the anterior infundibulo-trabecular junction (bold black arrow). **(C)** Diffuse anteroapical hypokinesia with anteroapical akinesia (four bold black arrows), inferior and sub-tricuspid hyokinesia (thin black arrow).

***Qualitative analysis***. The 26 patients from BrS group II (without overt structural abnormalities) had three different types of segmental wall motion abnormalities (Figure 4): (1) diffuse anterior RV hypokinesia or akinesia (*n* = 15), corresponding to an extended anteroapical RV hypokinesia, sometimes associated with an inferior RV hypokinesia (*n* = 9); (2) localized apical RV hypokinesia or akinesia (*n* = 7); and (3) hypokinesia or akinesia restricted to the anterior RV infundibulo-trabecular junction (*n* = 4). BrS group III patients (*n* = 10) had RV structural abnormalities (deep fissuring) corresponding to early or localized ARVD/C, predominantly affecting the RV anteroapical wall (Figures 1, 2).

LV anatomy was comparable between BrS groups I, II, and III. None of the patients had RV or LV microaneurysms (Frustaci et al., 2005).

***Quantitative assessment of rv systolic function (Table 3)***. The RVEF and TAPSE were comparable between BrS groups I, II, and III, as well as between BrS group and control patients. However, RVEF was significantly higher in BrS group compared to ARVD/C patients without ST segment elevation (57 ± 5 vs. 54 ± 5%, *p* = 0.006). This difference was related to a higher RVEF among patients with a BrS-ECG and no ARVD/C (BrS groups I+II, *n* = 41) compared to ARVD/C patients without ST segment elevation (58 ± 5 vs. 54 ± 5%, *p* = 0.004). RVEF was comparable between the BrS group III and ARVD/C patients without ST segment elevation (56 ± 5 vs. 54 ± 5%, *p* = NS).

Statistically significant differences in terms of segmental excursion between BrS groups I, II and III were seen for the anterior, apical (BrS group I vs. II, BrS group I vs. III) and inferior (BrS group I vs. II) walls of the RV. On one hand, there was no difference in terms of RV segmental excursion between BrS groups II and III. On the other hand, only localized ARVD/C (with or without BrS-ECG) provided anteroapical dyskinesia. The excursion of the mid-lateral wall was significantly larger in patients with a BrS-ECG and no ARVD/C (BrS groups I+II), as compared to control patients and ARVD/C patients without ST segment elevation (12.6 ± 3.3 vs. 10.3 ± 3.0 and 10.6 ± 3.8 mm, *p* = 0.02).

***Quantitative assessment of lv systolic function (Table 3)***. The LVEF was comparable between BrS groups I, II, and III, and between BrS group and control patients.

No complication occurred during follow-up.

### Genetics

The results of our genetic study (*n* = 45) are reported in Tables 1, 2. A missense R965C *SCN5A* mutation was found in one patient (patient 41, BrS group II), resulting in a single amino-acid substitution. A Black african male (patient 33) belonging to BrS group I carried an African *SCN5A* polymorphism (R481W) concerning a highly preserved gene segment among 5 species and 300 controls, which has been described in a recent publication as a special polymorphism (Tan et al., 2005). He had also a double polymorphism of *DSG2* and *DSC2*. Patient 37, native of Cambodia, carried a *TRPM4* mutation p.Q131H (c.393G>C) and had a baseline incomplete right bundle branch block, associated with a type 3 BrS-ECG and a left anterior fascicular block.

Among the 10 patients from BrS group III who underwent a more extensive genetic study, no desmosomal gene mutation was found. Patient 43 carried a rare *PKP2* polymorphism (missense, A264V) and patient 44 carried a *DSG2* polymorphism (p.Ser882Pro, c2644T>C). No *SCN5A* mutation was found in this group (Table 2, bottom). Another *PKP2* polymorphism was found in patient 24 (BrS group II).

## Discussion

BrS is an important cause of SCD among young people, particularly in men, like hypertrophic cardiomyopathy and ARVD/C [BrS represents 4 to 10% of all SCD and 20% of SCD with a normal heart (Antzelevitch et al., 2005)]. In their first report, Brugada and Brugada described eight cases without echocardiographic or angiographic abnormalities (Brugada and Brugada, 1992). In later studies however, the absence of structural abnormalities was presumed from routine echocardiography and MRI, which have a poor sensitivity for the diagnosis of focal ARVD/C, myocarditis (microaneurysms) or early stage of cardiomyopathy, all able to mimic a BrS (Frustaci et al., 2005). The main objective of the present study was to look for a potential overlap between BrS and ARVD/C by assessing prospectively their respective prevalence on the basis of international criteria, in patients with BrS-ECG and normal echocardiography. This question is important in daily practice when a patient is referred for BrS or BrS-ECG evaluation and management. Therapeutic options remain indeed challenging in the setting of a BrS-ECG pattern associated with ARVD/C as arrhythmias are usually triggered by exercise in ARVD/C and by vagal tone and rest in BrS, but also because anti-arrhythmic drugs such as all I_c_ class (flecainide) and beta-blockers are protective in ARVD/C while contra-indicated (class I_c_) or not logical in BrS (isoproterenol is useful for electrical storm management in BrS) (Postema et al., 2009).

### Main findings

In our series, the primary endpoint was achieved in 4/51 patients (8%) with a BrS-ECG who fulfilled both ESC/WHF 2000 ARVD/C criteria and HRS/EHRA 2005 BrS criteria, despite a normal echocardiography.

Among BrS group, 34/51 patients (67%) fulfilled BrS HRS/EHRA 2005 criteria, and 8/51 (16%) fulfilled ESC/WHF 2000 ARVD/C criteria. Contrast angiography showed RV wall motion abnormalities in 36/51 patients (71%). Among these patients, 26 (51%) had RV wall motion abnormalities without structural alterations (i.e., deep fissuring, BrS group II), and 10 (20%) presented structural alterations compatible with localized (anteroapical and/or diaphragmatic) ARVD/C (BrS group III). Only 15 (29%) patients had a strictly normal RV at angiography.

To our knowledge, this is the first study that showed prospectively an overlap between BrS and ARVD/C. This notion of an overlap may suggest that some patients of our series belonging to BrS group II (i.e., segmental wall motion abnormalities without ARVD/C) could present a potential or subclinical ARVD/C, as it is the case among BrS group III patients. In our opinion, three assumptions can be made to explain this overlap: (1) the existence of functional abnormalities of myocardial contraction due to impaired depolarization [i.e., late activation (Coronel et al., 2005; Nademanee et al., 2011) and incomplete right bundle branch block] and impaired repolarization associated with BrS [i.e., transmural voltage gradient due to loss of the epicardial action potential dome (Yan and Antzelevitch, 1999)]. This hypothesis could be applied in cases of diffuse RV hypokinesis (anteroapical and inferior) or in case of localized high RVOT (i.e., infundibulotrabecular junction) bulging seen on MRI or contrast angiography; (2) the second hypothesis relies on the fact that structural abnormalities (including fibrosis and fatty infiltration) have been reported in association with BrS and/or mutations in the cardiac sodium channel, as were able to demonstrate Coronel et al. (Coronel et al., 2005). This might be the case in the presence of segmental RV akinesia or concealed ARVD/C and could be attributed to an excitation failure of the myocardium, in particular epicardial; and (3) RV conduction disturbances observed in BrS may explain a delayed onset of RV contraction, besides increasing RV wall stress (current-to-load mismatch) which could be responsible of the RV structural abnormalities over time (Coronel et al., 2006; Hoogendijk et al., 2010).

### Electrophysiology

Ajmaline testing is recognized as the best method to unmask BrS-ECG. Its sensitivity, although imperfect, ranges from 80 to 100% among series (Priori et al., 2000; Hong et al., 2004; Wolpert et al., 2005), which is far better than with flecainide acetate (68%). For Hong et al. (Hong et al., 2004), ajmaline testing has a good sensitivity and PPV to identify *SCN5A* mutations (94.4 and 93.3%, respectively), making us confident in the diagnosis of BrS in our series.

Late potentials were present in 73% of the patients (27/37) of our BrS series. These results are concordant with Kim et al. series (82%) (Kim et al., 2006). Late potentials were present in 63% (17/27) of patients without ARVD/C (groups I and II) and in 60% (12/20) of patients without ARVD/C and fulfilling HRS/EHRA diagnostic criteria for BrS. Their presence does not appear specific of BrS with RV segmental wall motion abnormalities or of an organic RV disease like ARVD/C. Late potentials on SA-ECG have been related to the presence of epicardial diastolic (late) potentials located to the anterior wall of the RVOT (Nagase et al., 2002). For Antzelevitch et al., their presence could be the functional consequence of an epicardial notch accentuation associated with a loss of the action potential's dome (Antzelevitch, 2002), but other authors (Coronel et al., 2005) have shown the presence of a delayed endocardial conduction through the RVOT, in the absence of a transmural gradient, but in the presence of localized interstitial fibrosis, i.e., organic. These observations support the hypothesis of an arrhythmogenic substrate located to the RVOT. Indeed, Nademanee et al. have demonstrated that the arrhythmic underlying electrophysiological mechanism in patients with BrS was delayed (>100 ms) depolarization over the anterior aspect of the RVOT epicardium, showing low voltage (<1 mV), prolonged (>130 ms) and fractionated late potentials. Catheter ablation over this abnormal area resulted in normalization of the BrS-ECG and prevented further VT/VF recurrence (Nademanee et al., 2011). In our series, SA-ECG was useful to exclude the diagnosis of localized ARVD/C associated with a BrS-ECG, with a sensitivity of 100% and NPV of 100%, despite poor specificity and PPV (at 40 and/or 25 Hz, positive if ≥2 criteria).

The low rate of inducible patients during PVS in our series (28%), compared to previously published series reporting an inducibility ranging from 36 (Kim et al., 2006) to 67% (Priori et al., 2000), can be explained by the broad proportion of pauci-symptomatic patients in our series, but also by the poor sensitivity and reproducibility of this tool, which has been shown even in survivors of VF (Priori et al., 2000; Eckardt et al., 2005). The prognostic value of the PVS seems limited, particularly in patients at low or intermediate risk like in our series, since it is known that the association of a BrS type 1 ECG with a history of spontaneous syncope is the major determinant of prognosis in the BrS (Priori et al., 2000; Eckardt et al., 2005; Probst et al., 2010).

### Cardiac imaging and hemodynamics

The presence of angiographic morphological abnormalities in 71% of cases in this series confirms the relevance of a biventricular invasive study as part of the initial assessment of BrS, yet rarely achieved in this indication. Our results also emphasizes the inability of standard echocardiography to identify early stages of cardiomyopathies like localized ARVD/C, but also the limitations of cardiac MRI (Frustaci et al., 2005) or radionuclide angiography in these indications (false positives for MRI, lack of sensitivity for MRI and radionuclide angiography). However, Takagi et al. (Takagi et al., 2001) were the first to demonstrate *in vivo* morphological MRI abnormalities of the RV in BrS, showing hypokinesia, akinesia or localized aneurysms, which were present in 81% of cases. Recently, Catalano et al. found with MRI a high prevalence (50 vs. 17% for controls) of mild RV wall motion abnormalities among patient with BrS associated with a reduction in RVOT ejection fraction, an enlargement of the RV inflow diameter, and a mild increase in RVESV (not RVEDV) without fatty infiltration (Catalano et al., 2009). These data together with other observations support the fact that morphological and histological abnormalities are very common in BrS (Frustaci et al., 2005; Kim et al., 2006; Zumhagen et al., 2009). They may take the form of simple RV wall motion abnormalities, of moderate changes such as hypertrophy, mild fibrosis, and fatty infiltration, but also of genuine early or localized cardiomyopathies (documented by endocardial biopsies by Frustaci et al.), which may include ARVD/C, myocarditis or a latent form of cardiomyopathy.

With quantitative digitized contrast angiography, we confirmed *in vivo* the postmortem observations of Corrado et al. (Corrado et al., 1996, 2001) which showed that the clinical forms of ARVD/C associated with a Brugada ECG were exclusively localized or focal, affecting the antero-apical wall of the RV and taking the aspect of a predominantly fatty ARVD/C. Moreover, the hemodynamic profile of our patients and the functional angiographic analysis suggest that ARVD/C associated with a BrS-ECG may correspond to an earlier phase of ARVD/C disease as compared to localized forms of ARVD/C without ST segment elevation in the right precordial leads.

### Genetic study

The prevalence of a *SCN5A* mutation in our series was particularly low [2.2% in our series, as compared to 15–25% in the literature (Ackerman et al., 2011)]. This might be explained by the low prevalence of family history of BrS/SCD in our series (12/51, 23%). However, BrS-ECG was well characterized. This could also be due to the fact that the population under study is made of patients with a BrS-ECG pattern, without evidence for an overt structural heart disease (on echocardiogram), but which may include early forms of cardiomyopathies like ARVD/C, myocarditis, both mimicking BrS-ECG, or pure BrS.

One patient was initially thought to have a R481W mutation that has proven to be retrospectively a rare African polymorphism. However, this particular polymorphism could be not benign as its presence induces *in vitro* sodium current altered electrophysiology. Tan et al. showed (Tan et al., 2005) that the R481W polymorphic channel found in Blacks and Hispanics produced a significantly slower sodium current recovery from inactivation in both splice variant backgrounds and increased slow inactivation in Q1077del background compared with wild-type. Also, R481W showed a significantly negative shift of inactivation midpoint in Q1077del background. Shifts of a similar magnitude have been reported for arrhythmia syndrome mutations and are thought to be pathogenic.

Candidate gene analysis for *MOG1* was disappointing as no patient out of 41 screened carried a mutation, even if it has been showed that dominant-negative mutations in *MOG1* can impair the trafficking of Na(v)1.5 to the membrane, leading to *I*_Na_ reduction and clinical manifestation of BrS (Kattygnarath et al., 2011). The presence of a previously published *TRPM4* mutation (patient 47, p.Q131H, c.393G>C) suggests a relationship between progressive conduction diseases and BrS (Stallmeyer et al., 2012).

Given our analysis of genes encoding proteins of the cardiac desmosome in patients with BrS and RV structural abnormalities compatible with ARVD/C, we would not recommend to test *PKP2*, *DSP*, *DSG2*, and *DSC2* genes routinely in BrS. However, genetic testing is negative in 30–70% of overt forms of ARVD/C (Ackerman et al., 2011) and new candidate genes implicated in ARVD/C pathogenesis could be still good candidates for genetic screening in BrS. The absence of gene mutation in our patients with BrS-ECG and ARVD/C could also suggest the existence of a new entity, distinct from pure BrS and from ARVD/C itself, which could be genetic or acquired (such as myocarditis sequelae for example).

Our results underline the limited role of a systematic genetic screening in BrS and the debated causative role of *SCN5A* mutation in BrS (Coronel et al., 2006). Genetic testing has been recently recommended by HRS/EHRA experts for family members and appropriate relatives following the identification of the BrS-causative mutation in an index case; *SCN5A* mutation screening can be useful for any patient in whom a cardiologist has established a clinical index of suspicion for BrS; genetic testing is not indicated in the setting of an isolated type 2 or 3 BrS-ECG (Ackerman et al., 2011). Nevertheless, pathophysiology of BrS is complex and some authors as Coronel et al. and Frustaci et al. have reported three authentic *SCN5A* mutations associated with histological and/or angiographic findings consistent with ARVD/C in patients with BrS (Coronel et al., 2005; Frustaci et al., 2005), raising again the question of an overlap between BrS and localized forms ARVD/C (Perez Riera et al., 2005; Hoogendijk et al., 2010).

### Study limitations

Given that quantitative contrast angiography remains a gold-standard in ARVD/C, we do not currently have another available technique to assess the diagnostic accuracy of contrast angiography, apart from the histological examination of endomyocardial biopsies. These biopsies were not performed in the present series for safety reasons and for their poor diagnostic yield.

Genetic analysis was incomplete in this series as 6 patients without ARVD/C (6/41) refused genetic screening and as only 4 patients without ARVD/C underwent a finally negative screening of *PKP2*, *DSP*, *DSG2*, and *DSC2* genes. Patients with ARVD/C and BrS may also present large genomic deletions, large insertions or genomic rearrangement, as we did not dispose at the time of the study of Multiplex Ligation-dependent Probe Amplification (MLPA) technique.

## Conclusions

In our series, 71% of the 51 consecutive patients with a BrS-ECG and a normal echocardiography had abnormal RV wall motion and 16% had structural alterations corresponding to localized (anteroapical and/or diaphragmatic) ARVD/C at angiography. Moreover, 8% of patients with a BrS-ECG fulfilled both BrS and ARVD/C international criteria. Our results strongly support the hypothesis of an overlap between BrS and localized or concealed forms of ARVD/C, but also that Brugada ECG pattern is not specific of a pathophysiological entity, i.e., BrS, but can be due to other conditions such as myocarditis or localized RV cardiomyopathy.

In patients with such an overlap, and based on our experience, we would recommend to avoid Class Ic antiarrhythmic drugs prescription, automedication and fever (by the use of paracetamol or aspirin) as for any BrS patient. If ventricular arrhythmias are present, hydroquinidine and/or beta-blockers use could be considered, under careful ECG and Holter-ECG monitoring. ICD implantation indications should follow current available guidelines.

We believe that a BrS ECG pattern may be an indicator of ARVC, particularly in its clinically concealed phase, thus requiring morphological RV evaluation, such as MRI, contrast echocardiography and/or contrast angiography. One of the explanations of the low prevalence of SCN5A mutations in our series may lie in the high proportion of structural abnormalities and of localized ARVD/C. Several reasons may explain that difference as compared to other series: (1) most of the Brugada series did not assess the RV morphology with more than a standard echocardiogram, which is of particularly limited interest for RV study; (2) very few authors performed digitized contrast angiography in BrS; and (3) among the 10 patients who happened to have RV morphological abnormalities compatible with ARVD/C, none presented a desmosomal mutation, which is atypical. This overlap between BrS and ARVD/C does not mean that these two diseases are the same one, but that a substantial proportion of patients with a Brugada ECG pattern present RV structural alterations.

### Conflict of interest statement

The authors declare that the research was conducted in the absence of any commercial or financial relationships that could be construed as a potential conflict of interest.

## Abbreviations

BrS: Brugada Syndrome
BrS-ECG: Brugada Syndrome ECG pattern
SCD: Sudden Cardiac Death
VF: Ventricular Fibrillation
ARVD/C: Arrhythmogenic Right Ventricular Dysplasia/Cardiomyopathy
RV/LV: Right/Left Ventricle
PPV/NPV: Positive/Negative Predictive Values
RAO/LAO: Right/Left Anterior Oblique (projection)
EF: Ejection Fraction
TAPSE: Tricuspid Annulus Plane Systolic Excursion
SA-ECG: Signal Averaged-Electrocardiogram
PVS: Programmed Ventricular Stimulation
RVOT: Right Ventricular Outflow Tract
VT: Ventricular Tachycardia
MRI: Magnetic Resonance Imaging
EDV_i_/ESV_i_: End Diastolic Volume index/End Systolic Volume index
SI: Stroke Index.
